# The α-synuclein proteostasis network and its translational applications in Parkinson’s disease

**DOI:** 10.1073/pnas.2513317123

**Published:** 2026-03-16

**Authors:** Christine M. Lim, Michele Vendruscolo

**Affiliations:** ^a^Centre for Misfolding Diseases, Yusuf Hamied Department of Chemistry, University of Cambridge, Cambridge, Cambridgeshire CB2 1EW, United Kingdom

**Keywords:** protein homeostasis, Parkinson’s disease, alpha-synuclein

## Abstract

Parkinson’s disease (PD) is a debilitating neurodegenerative condition that results in a loss of mobility and muscle control. A neuropathological hallmark of PD is the presence of aberrant inclusions, known as Lewy pathology, of which α-synuclein (α-Syn) is a major component. The accumulation of α-Syn may be due to an imbalance in the proteostasis system regulating α-Syn. To investigate this hypothesis, we delineated the proteostasis network (PN) of α-Syn in the human *substantia nigra* at the proteomic and transcriptomic level. We then defined an α-Syn proteostasis activity score (PAS) that quantifiably describes the relative activity of the α-Syn PN in promoting or inhibiting α-Syn aggregation. We report a corresponding PAS signature indicative of disease state, age-of-death in PD patients, and brain regional vulnerability to α-Syn aggregation in PD and healthy brains. After establishing the relevance of our network to PD, we developed a transcriptome-derived network model as an operational digital twin of the α-Syn PN in human *substantia nigra* cells from single-cell data and used it to prioritize candidate targets for PD. We then further showed the application of the α-Syn PN toward facilitating drug repurposing. Overall, this proof-of-concept study illustrates how our computational framework can identify and prioritize putative therapeutic targets and repurposing candidates for PD, providing testable hypotheses for experimental validation.

## Significance

As proteostasis collapse drives many neurodegenerative disorders, it is important to develop quantitative frameworks for its assessment. Here, we map the α-synuclein (α-Syn) proteostasis network in the human *substantia nigra* by integrating transcriptomic and proteomic data. We thus derive a proteostasis activity score (PAS) that captures the balance between aggregation-promoting and aggregation-attenuating pathways. PAS stratifies Parkinson’s disease (PD) vs. control brains, correlates with age at death, and predicts regional and peripheral vulnerability to pathology. Network influence analyses highlight previously unrecognized master regulators, six actionable targets, and 28 approved drugs that shift the network toward α-syn clearance. Our work provides a generalizable blueprint for decoding proteostasis landscapes and accelerating therapeutic discovery across protein-misfolding diseases with potential applications in precision medicine.

Parkinson’s disease (PD) is a neurodegenerative condition that progressively causes debilitating motor symptoms and cognitive impairment (1–7). Despite affecting over 6 million people globally (1–7), its molecular origins remain incompletely understood, and no disease-modifying treatments are currently available in the clinic (8).

The aggregation of α-synuclein (α-Syn) into Lewy bodies (9, 10) is a molecular hallmark of PD (1–7), a feature that can be leveraged for the development of quantitative diagnostic methods (11, 12) and for the design of clinical trials (13). It has been proposed that α-Syn aggregates, whether small oligomers, protofibrils, or mature fibrils, may be neurotoxic (14–17) making α-Syn a primary target for therapeutic interventions (8, 18). The failure to maintain α-Syn in its functional state indicates that the cellular mechanisms responsible for the removal of damaged or misfolded forms of α-Syn are impaired in PD. These mechanisms are part of the protein homeostasis (proteostasis) network (PN), which regulates the behavior of proteins in terms of their conformations, interactions, concentrations, and localizations (19, 20). A defective PN has been associated with aging and increased vulnerability to disease (21–23), suggesting that the multifactorial nature of PD is linked with the specific impairment of PN subsystems.

In this work, we mapped the PN of α-Syn, the subsystem of the overall PN that is specifically concerned with α-Syn regulation, to investigate the hypothesis that disruptions in the balance of the α-Syn PN contribute to the accumulation of α-Syn in PD. We implemented this approach by first identifying components of the α-Syn PN using functional protein interactions and transcriptomic analysis of six PD microarray datasets. We then validated our findings across disease progression in an independent PD dataset. Based on this α-Syn PN, we computed an α-Syn proteostasis activity score (PAS) to quantitatively describe whether the network is promoting or inhibiting α-Syn aggregation under various conditions. We reported that PAS is indicative of disease states, age of death, and regional vulnerability in PD patients and healthy cohorts. Finally, we further showed how the α-Syn PN can be used for prioritizing targets and facilitating drug repurposing, thereby acting as a proof-of-concept for our informatics approach designed to rapidly identify high-value candidates for α-Syn proteostasis regulation, offering an initial screen to direct and accelerate downstream wet-lab investigations.

## Results

### Building the α-Syn PN.

**Fig. 1.**
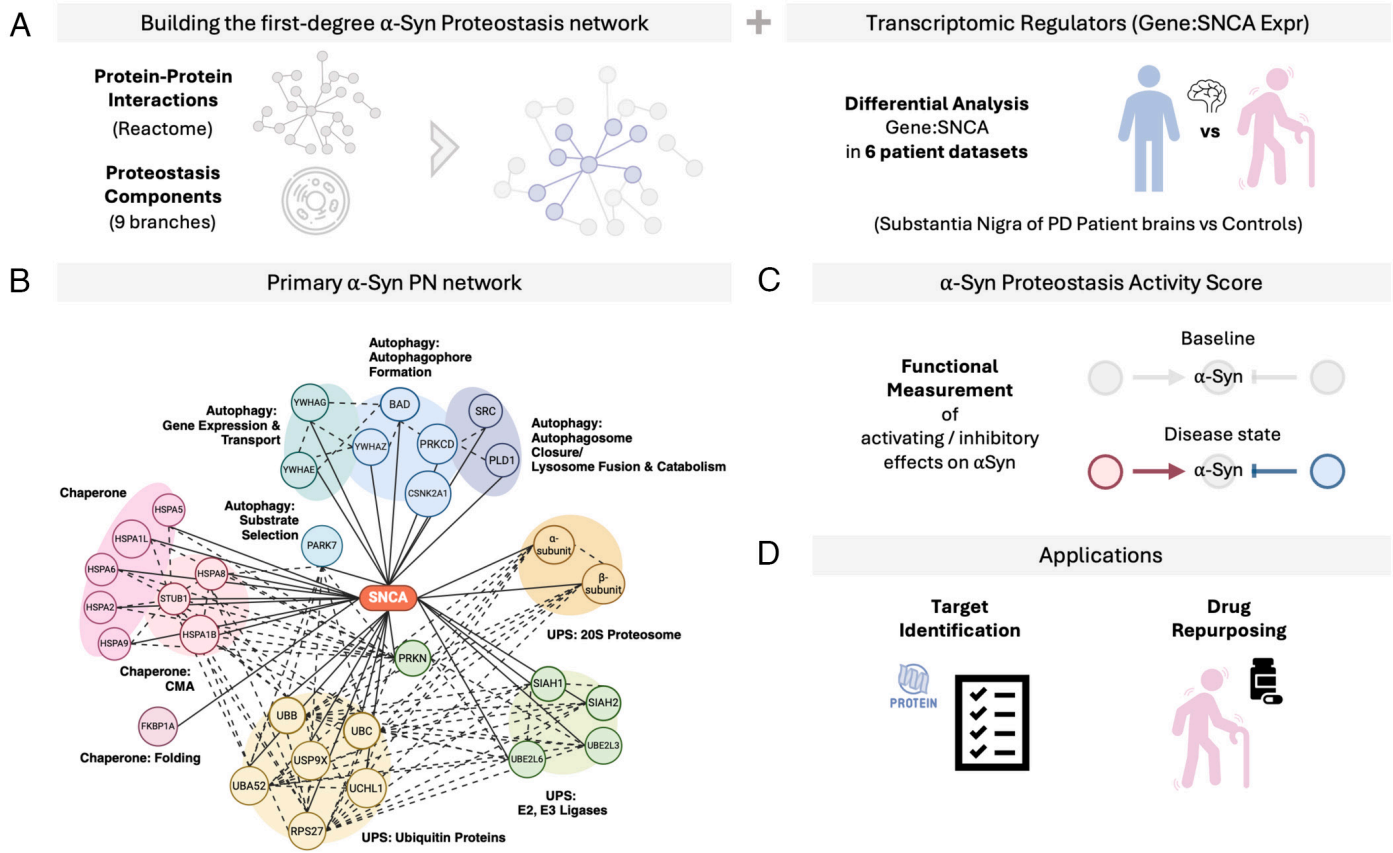
Study design and objectives. (*A*) We assembled the α-Syn PN by collecting first-degree α-Syn interactors and classifying each as an aggregation promoter or attenuator based on the literature (Dataset S1). (*B*) We expanded the PN by adding genes differentially perturbed relative to *SNCA* in PD brains, yielding the final α-Syn PN. First-degree regulators are organized into three degradation pathways: CMA, the UPS, and the ALP. (*C*) We quantified PN activity on α-Syn with the PAS, which captures PD status, age at death, and regional vulnerability. (*D*) We illustrate how the α-Syn PN and PAS enable target prioritization and drug repurposing analyses.

We first delineated the α-Syn PN at the proteomic and transcriptomic level. To do this, we started by identifying the primary (first-degree) α-Syn protein interactors within the overall PN (*Methods* and Fig. 1*A*) and classified them into promoters and attenuators of α-Syn aggregation based on literature reports (Dataset S1). To further extend the PN of α-Syn, we identified the genes perturbed relative to *SNCA* in PD brains (Fig. 1*A*). This analysis was based on the hypothesis that the balance in the expression of α-Syn relative to proteins involved in maintaining α-Syn proteostasis is perturbed in PD, resulting in α-Syn accumulation. To investigate this possibility, we carried out differential expression analysis of PD vs. control *substantia nigra* samples in 6 microarray datasets (*Methods*). To account for the balance of each gene to *SNCA*, all genes were normalized to *SNCA* expression by calculating log2(gene)-log2(*SNCA*) for differential expression quantification. Based on our analysis, 108 genes were consistently altered (directionality) in at least half of the datasets analyzed (Dataset S2). To integrate these genes into the existing α-Syn PN generated from protein–protein functional interactions above, we analyzed the functional interactions between each of the 108 genes (whose expression is consistently altered with respect to *SNCA*) with the existing primary first-degree PN interactors of α-Syn. In this way, 21 genes were found to encode proteins that have functional interactions with the first-degree primary α-Syn PN established earlier (Datasets S3 and S4). To validate the perturbation patterns of these 21 genes in PD, we analyzed a separate microarray dataset (*Methods*) consisting of *substantia nigra* samples from PD brains of different Braak stages (9, 24) (*SI Appendix*, Fig. S1), finding that the same trends of perturbation are retained. To benchmark the identification of gene markers via computing gene:*SNCA* relative expression changes, we studied the known PD genes LRRK2 and PINK1, finding that their relative expressions are indicative of brain regional vulnerability in healthy samples. In contrast, genes identified just via differential expression (25) may not always be relevant when considered in relation to α-Syn (*SI Appendix*, Fig. S2).

### The α-Syn PN.

The α-Syn PN is presented in Fig. 1*B*. The majority of the α-Syn PN is made up by molecular chaperones and by degradation systems, including chaperone-mediated autophagy (CMA), the ubiquitin–proteasome system (UPS), and the autophagy-lysosome pathway (ALP). This structure of the α-Syn PN is consistent with previous reports indicating that a reduced ability to degrade damaged or misfolded α-Syn is a driver of α-Syn aggregation in PD and related synucleinopathies (26–28), and it represents a possible therapeutic target (8, 18, 29).

CMA is one of the main pathways to remove potentially cytotoxic excessive amounts of α-Syn in PD (26). In the degradation of α-Syn via CMA, α-Syn is recognized by cytosolic chaperones and transported into lysosomes where it is degraded by proteases (30, 31). In familial PD, mutant forms of α-Syn are poorly degraded via CMA as mutant α-Syn binds CMA receptors resulting in a blockage of CMA degradation (26). In a similar fashion, downregulated CMA activity due to other environmental circumstances may reduce α-Syn clearance promoting buildup in sporadic cases of PD. UPS is also a major degradation pathway for α-Syn clearance where dysfunctional UPS may contribute to rising levels of α-Syn in PD neurons (32). This possibility is supported by reports of reduced rates of proteasome catalytic activity (27) and lower levels of proteasome subunits in PD brains compared to healthy controls (33). Furthermore, the inhibition of the UPS was found to trigger PD neuropathology (34, 35). Given these findings, an impairment of the UPS is a likely contributing factor to α-Syn aggregation in PD.

ALP also plays an essential role in α-Syn degradation, and it is necessary for preventing PD-related α-Syn aggregation (32). Inhibition of autophagy increases α-Syn accumulation and aggregation, while its activation promotes the clearance of α-Syn inclusions (36, 37). Temporal changes in α-Syn accumulation have also been observed in accordance with changes in key ALP markers (36).

In the following sections, we used the α-Syn PN to measure its overall activity on α-Syn (Fig. 1*C*), and applied it for target prioritization and drug repurposing (Fig. 1*D*).

### The α-Syn PN Activity Is Indicative of PD States and Regional Vulnerability.

**Fig. 2.**
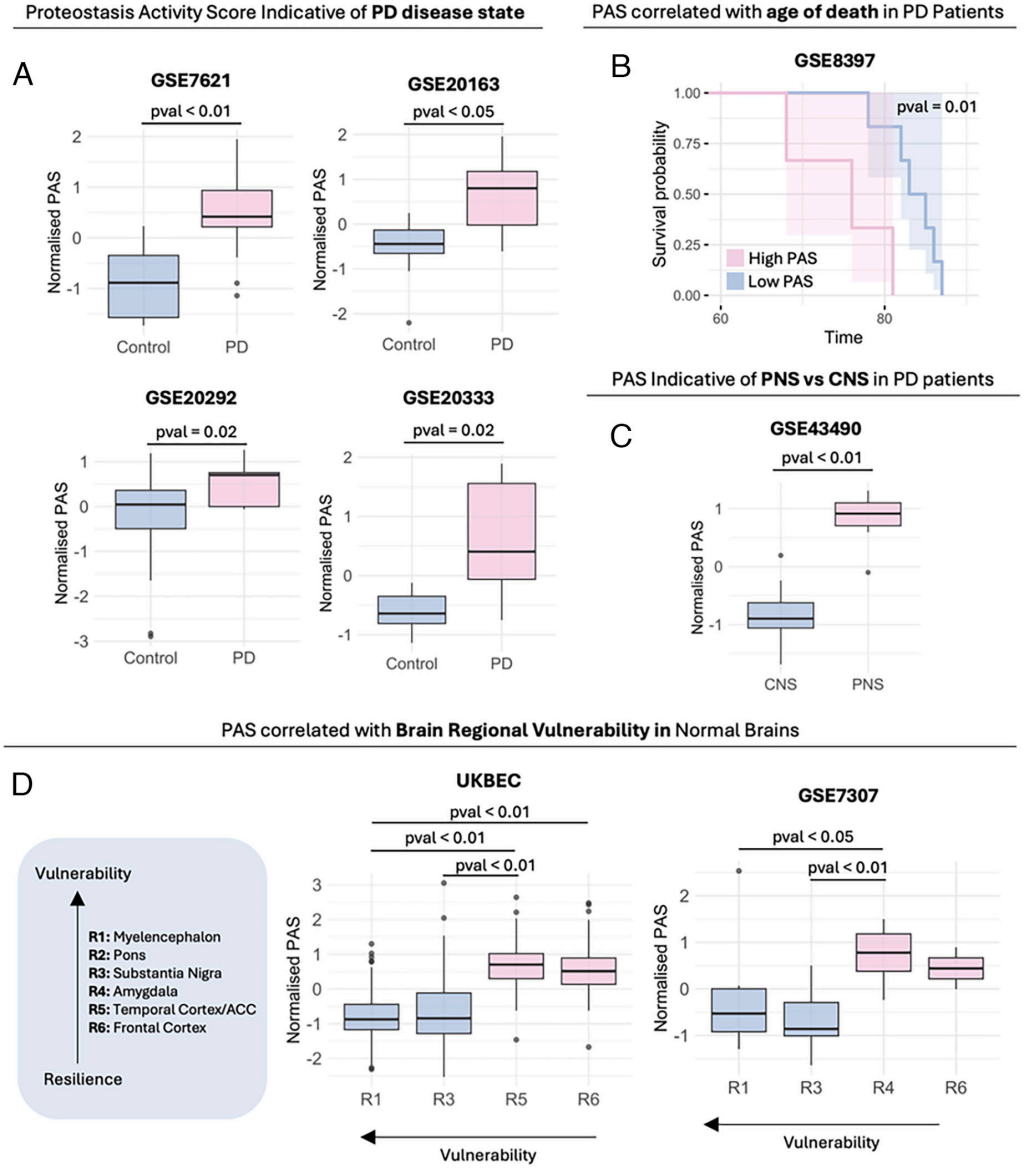
PAS stratifies disease status, mortality risk, and regional vulnerability. (*A*) PAS is elevated in PD brains relative to controls. (*B*) Within PD, higher PAS associates with a younger age at death. (*C*) In PD, regions that exhibit earlier α-Syn aggregation (vagus nerve and PNS) show higher PAS than CNS regions. (*D*) In healthy brains, baseline PAS inversely tracks regional vulnerability: More vulnerable regions display lower baseline PAS than more resilient regions. Statistical significance was assessed with the Wilcoxon test.

To capture the functional biological activity of the α-Syn PN, we defined PAS to capture the coordinated activity of the proteins within our network under different states. Details of the calculation of PAS are described in *Methods*. We found that PAS is indicative of disease state, as PD patients across four unique datasets have a higher PAS than controls (Fig. 2*A*), reflecting increased activity in promoting α-Syn aggregation compared to controls. In addition, PD patients with higher PAS were associated with a younger age of death (Fig. 2*B*). In patients with PD, the peripheral nervous system (PNS) has higher PAS than the central nervous system (CNS) (Fig. 2*C*) reflecting the higher vulnerability of the vagus nerve to be compromised to α-Syn aggregation in PD progression.

Two independent datasets [UKBEC (38) and GSE7307] including samples from multiple brain regions of nondisease patients were also analyzed to study regional vulnerability. We found that brain regions of higher vulnerability had lower baseline levels of proteostasis activity (Fig. 2*D*). These findings suggest lower basal activity in α-Syn aggregation promotion or higher inhibitory activity of α-Syn aggregation.

### Target Identification for PD.

**Fig. 3.**
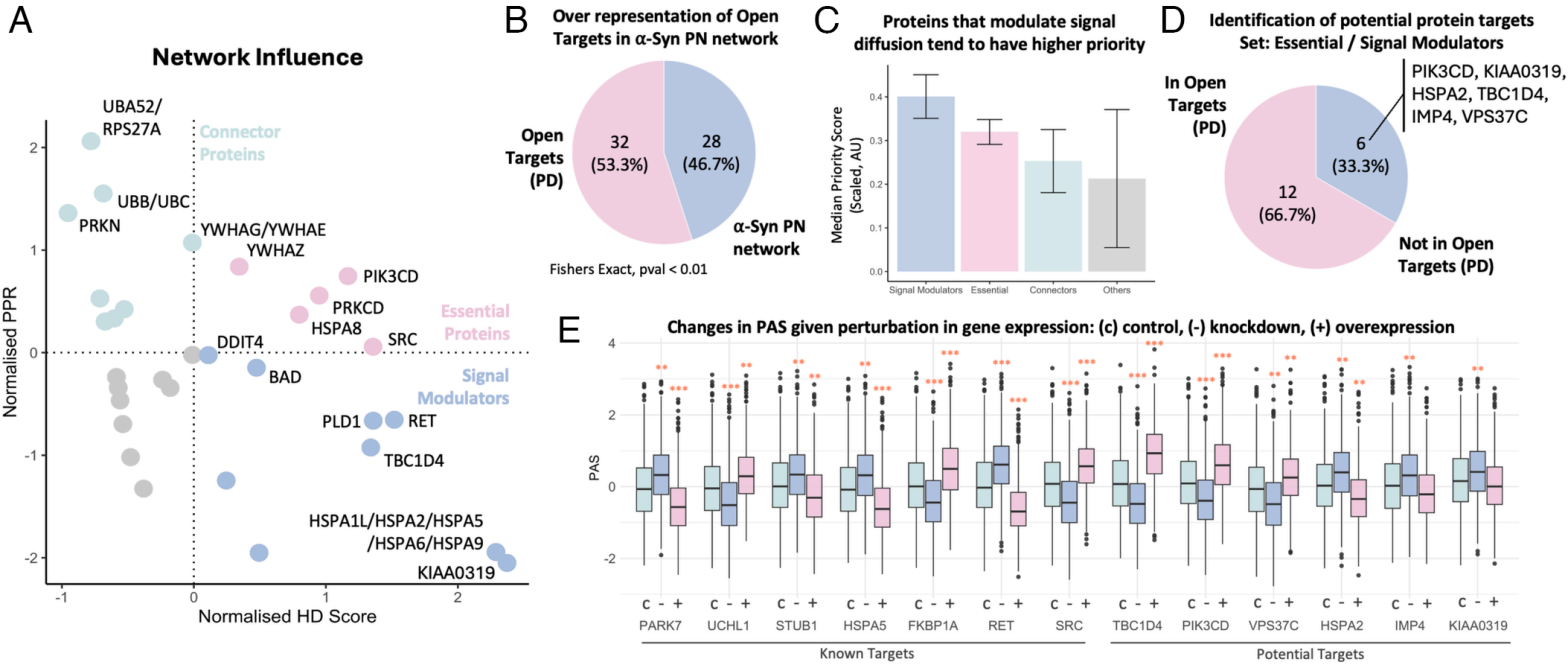
Network influence prioritizes candidate targets in the α-Syn PN. (*A*) We quantified the network influence of each protein using two complementary measures, heat diffusion and PPR. Proteins high in both heat diffusion and PPR act as master regulators (centrally connected and efficient propagators). Proteins with high diffusion but lower PPR propagate signals locally (specialized modulators), whereas proteins with high PPR but lower diffusion are well connected but less efficient propagators. (*B*) Of the 60 α-Syn PN proteins, 33 (55%) are annotated as PD targets in Open Targets. (*C*) Signal modulators and essential PN proteins (those with higher diffusion and modulation capacity) tend to receive higher PD priority scores in Open Targets. (*D*) Six of the 18 essential/signal modulators have not yet been reported as PD targets: PIK3CD, KIAA0319, HSPA2, TBC1D4, IMP4, and VPS37C. (*E*) In silico perturbations (activation/inhibition; *Methods*) indicate that modulating these targets can beneficially shift PN activity as quantified by PAS. Effect sizes were summarized with Cohen’s *d*: (*) d > 0.5; () *d* > 0.3.

To illustrate the use of the α-Syn PN to propose potential protein targets for PD, we hypothesized that proteins that exert larger influence on the α-Syn PN would be more likely to be relevant targets. For this purpose, we adopted two network influence scores—heat diffusion (39) and personalized page rank (PPR) (40). Heat diffusion quantifies the signal spread of a protein across the network to understand its influence over signal propagation in the network. The proteins are plotted on a 2-axis graph in Fig. 3*A*. Proteins with high heat diffusion and high PPR are likely to be master regulators, as they are central hubs and efficiently propagate signals through the network. Proteins with high heat diffusion and lower PPR efficiently, although not major hubs, propagate signals locally and likely modulate specialized functions within the network. Proteins with high PPR but lower heat diffusion are well connected but are less efficient at propagating signals through the network. 55% of the proteins making up the α-Syn PN have been identified to be target proteins for PD in the Open Targets database (Fig. 3*B*). Among these targets, signal modulators and essential proteins with higher heat diffusion and signal modulation capabilities tend to have relatively higher priority scores for PD as per Open Targets (Fig. 3*C*). Notably, 33% of our essential and signal modulators are proteins not yet reported as targets for PD (Fig. 3*D*): PIK3CD, KIAA0319, HSPA2, TBC1D4, IMP4, and VPS37C. Experimental inhibition of the target proteins beneficially modulates α-Syn PN activity as quantified by PAS (Fig. 3*E*).

To examine how candidate targets modulate α-Syn PN activity, we performed in silico perturbations of selected known and putative targets. We then constructed an operational digital twin, as a cross-sectional network model of the α-Syn PN, to approximate the molecular context of human *substantia nigra* cell populations. Built from aggregated single-cell transcriptomic data without cell-type stratification, the model supports exploratory inference of network-level changes under defined perturbations. Importantly, it learns partial-correlation structure from transcript levels and should be viewed as a hypothesis-generating surrogate, rather than a fully mechanistic, time-resolved, or individualized digital twin (41, 42). Details of the simulations are provided in *Methods*. Benchmarking our simulation to known targets (Fig. 3*E*), we found that downregulation of α-Syn aggregation-attenuating PARK7 results in a relatively higher PAS, indicating a shift of the α-Syn PN activity toward promoting α-Syn aggregation. Overexpression of α-Syn aggregation-promoting UCHL1 also increases the α-Syn PN aggregation-promoting activity while taking a more inhibitory slant upon downregulation. α-Syn aggregation-inhibiting STUB1 and RET shifts the α-Syn PN activity toward inhibiting α-Syn aggregation upon upregulation while increasing the aggregation-promoting activity upon their downregulation. Our top three candidate targets based on their effect size on the α-Syn PN given expression perturbations are TBC1D4, PIK3CD, and VPS37C (Fig. 3*E*). These three targets are upregulated relative to *SNCA* in PD and are predicted to promote the activity of the α-Syn PN toward α-Syn aggregation upon overexpression. In contrast, downregulating these targets is predicted to shift the α-Syn PN closer toward α-Syn inhibition. These findings suggest that inhibition of TBC1D4, PIK3CD, and VPS37C may be useful for managing the shift of the α-Syn PN toward promoting aggregation.

### Drug Prioritization for Repurposing.

**Fig. 4.**
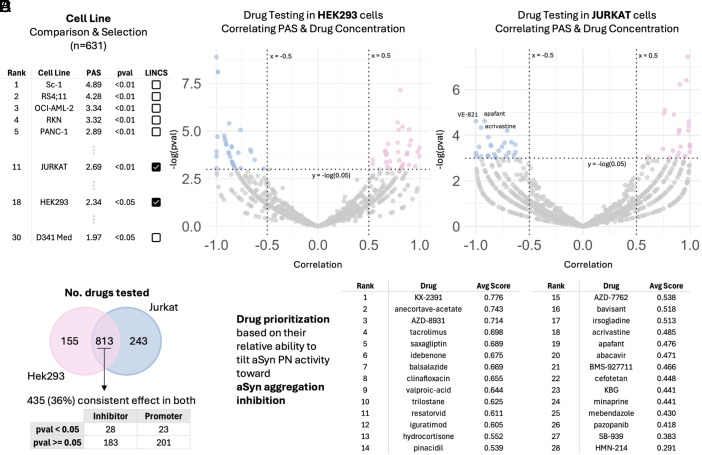
PAS-based quantification of drug responses in HEK293 and Jurkat cells identifies and prioritizes inhibitors of the α-Syn PN and aggregation. (*A*) Model selection: From 631 HPA cell lines with detectable *SNCA* (nTPM > 2), we ranked baseline PN activity by PAS (higher = more aggregation-promoting). Jurkat (rank 11) and HEK293 (rank 18) were selected based on availability in LINCS. For each line, differential expression was obtained for small-molecule treatments vs. DMSO. (*B* and *C*) For every compound, we computed the change in PAS (ΔPAS) after 24 h across increasing concentrations and calculated the concentration-ΔPAS correlation in HEK293 (*B*) and Jurkat (*C*). (*D*) Of 968 compounds tested in HEK293 and 1,056 in Jurkat, 813 were common to both; 435 of these showed concordant PAS effects across cell lines. Notably, 28 compounds acted as PN inhibitors (negative concentration-ΔPAS correlation in both), with *P*-value < 0.05 in at least one line. (*E*) To facilitate PD repurposing, the 28 inhibitors were prioritized by the mean absolute correlation across HEK293 and Jurkat.

We then investigated the use of the α-Syn PN as an evaluation metric to help identify and prioritize candidate drugs for PD. For this, we first selected cell models that best captured the shift in the α-Syn PN toward aggregatory activity. A comparison of 631 cell lines listed in the Human Protein Atlas (HPA) was ranked based on their relative PAS activity (Fig. 4*A*). Cell lines with higher PAS indicating α-Syn PN activities promoting α-Syn aggregation were given higher priority. From here, two model cell lines available in LINCS (a large-scale drug testing dataset) were identified: Jurkat (rank 11) and HEK293 (rank 18). Corroboration with literature revealed that both HEK293 (43–45) and Jurkat cells (46, 47) are relevant for PD-related studies. Differential gene expression data for both cell lines treated with various small molecules (968 drugs tested in HEK293 cells and 1,056 drugs tested in Jurkat cells) compared against matched cells treated with DMSO were obtained. Changes in PAS (ΔPAS) upon treatment were quantified for each drug at each concentration tested (treatment for 24 h). For each drug, we then calculated the correlation of increasing levels of drug concentrations with ΔPAS in both HEK293 (Fig. 4*B*) and Jurkat (Fig. 4*C*) cells. 813 drugs were tested in both cell lines, with 435 of those drugs exhibiting consistent effects on PAS in both cells (Fig. 4*D*). We highlight 28 inhibitors of α-Syn PN that significantly shift the α-Syn PN activity toward inhibiting α-Syn aggregation. To facilitate drug repurposing for PD, we prioritized the 28 inhibitory drugs according to their average absolute correlation score across HEK293 and Jurkat cells (Fig. 4*E*).

This method enabled the identification of PD-relevant drugs. Approximately 30% of the candidate drugs identified here have already been studied and reported to be beneficial in PD models. Tacrolimus, originally designed for preventing posttransplant organ rejection, was reported to lead to improvements in the functional features of dopaminergic neurons and behavioral phenotypes affected in PD at low doses in mice (48). Saxagliptin, originally an antidiabetic drug, was reported to significantly improve motor performance, muscle coordination and correct akinesia in rat PD models (49). Due to its antiparkinsonian efficacy, Saxagliptin was proposed as a novel approach toward the management of PD (49). Idebenone, originally prescribed for dementia, was reported to improve motor dysfunction, learning and memory (50), as well as reduce neuroinflammation (51) in PD mice models. Hydrocortisone, usually prescribed to relieve inflammation, prevents dopaminergic cell death in a PD cell line model (52). Pinacidil, usually used to control blood pressure, was found to exhibit anti-inflammatory effects in an in vitro PD microglia model (53). Bavisant, originally developed to treat ADHD, has completed phase 2 trials to treat PD-related symptoms. Minaprine has been identified as an antiparkinson drug in ChEBI (https://www.ebi.ac.uk/chebi/searchId.do?chebiId=51038). Pazopanib, first FDA-approved for treating advanced renal cell carcinoma, has anti-inflammatory and neuroprotective effects in dopaminergic neurons in mouse models (54).

## Discussion

Aberrant proteostasis is a defining feature of PD, as impaired degradation of protein aggregates leads to their accumulation and drives toxic gain-of-function effects that contribute to disease onset and progression (26–29, 32–37, 55). This hypothesis is consistent with the correlation between α-Syn aggregate burden and PD progression (7, 9, 11, 12) and extensive association of proteostasis dysregulation in neurodegenerative diseases (19–23). Building on these observations, we reported a proof-of-principle study for a computational framework that defines the PN of α-Syn and uses it to identify candidate therapeutic targets and candidate drugs.

We delineated the α-Syn PN using both proteomic and transcriptomic data. Components of our α-Syn PN were validated by literature and across Braak staging. The delineation of the α-Syn PN allowed for the study of disease states in PD patients, age-of-death, and brain vulnerability toward α-Syn aggregation in PD. To quantitatively describe the α-Syn PN activity and its effects on α-Syn aggregation promotion or attenuation, we defined a PAS, finding that this score effectively represents disease states. States with higher PAS (promoting α-Syn aggregation) are associated with PD, with a younger age-of-death among PD patients, and with the PNS, which is susceptible earlier in PD compared to the CNS. We found that in nonneurological brain samples, brain regions more vulnerable to PD tend to have lower basal PAS than relatively more resistant brain regions. These observations may be linked to the threshold theory of PD (56) and are consistent with studies showing that intrinsic, region-specific proteostasis signatures in healthy brains anticipate tissue vulnerability (21, 23, 57, 58). In this view, PD is a systemic disease where α-Syn accumulation starts simultaneously in various brain regions but develops α-Syn pathology at different stages due to differential tissue-specific vulnerability. This hypothesis is also consistent with our findings in nonneurological samples, as well as previous studies finding lower basal levels of *SNCA* in more vulnerable brain regions compared to more resilient regions (25). Our results suggest that regions that have a higher susceptibility toward α-Syn aggregation (lower threshold: Lower basal levels of PAS/*SNCA*) present disease features earlier than regions with higher resilience toward α-Syn aggregation (higher threshold: higher basal levels of PAS/*SNCA*). Overall, the definition of PAS offers a metric that can be widely applied to describe the functional state of the α-Syn PN.

We next demonstrated how the α-Syn PN, integrated with a digital twin model of the α-Syn PN in human *substantia nigra* cells, can be leveraged to guide and prioritize experimental efforts in target identification. For this purpose, we built a static digital twin of human *substantia nigra* cells to capture protein–protein dependencies of the α-Syn PN in the context of the human *substantia nigra*. This approach offers two key benefits. The first is that digital twinning allows for the simulation of complex processes to study network responses to perturbations such as knockdown, overexpression, and drug perturbations. By doing so, we can perform a first screen and filter the targets channeled into experimental stages, thus saving time, effort, and costs. Second, digital twins of hard-to-access tissues, such as those in the brain, which can often only be sampled postmortem, enable us to study complex biological responses in these environments that are more relevant to the disease tissue, as compared to standard drug testing cell lines. By applying a digital twin of the α-Syn PN in human *substantia nigra*, we showed that simulated down-/up-regulation of benchmark genes (e.g., PARK7, UCHL1, STUB1, RET) modulates PAS in the expected directions, and that our top candidates (TBC1D4, PIK3CD, VPS37C) produce large, directionally consistent PAS shifts in silico (Fig. 3*E*). While this technology is still in its early stages, we highlight its potential in enabling simulated drug testing directly in disease-relevant tissues. We anticipate that this concept will enable progress in facilitating translational applications and improve patient outcomes.

In a second application, we investigated drug repurposing based on measuring changes of PAS in response to drug treatment. This analysis was done in HEK293 and Jurkat cells and yielded 28 drugs predicted to shift the α-Syn PN activity toward α-Syn aggregation inhibition. Eight of these 28 drugs have been reported to be associated with use for PD, reflecting the ability of our framework to prioritize drug targets.

Together, the delineation of the α-Syn PN, the correlation of PAS with key disease features, and the identification of PD-relevant candidate targets serve as internal validation of our approach. The ability of our method to capture effects of benchmark PD genes and drugs provides confidence that we can extract biologically relevant candidates. The six prioritized gene targets and 28 potentially repurposable drugs are candidates for future experimental validation, bridging the gap between computational discovery and translational research. For instance, our prioritization logic in Fig. 3*E* guides the validation of gene candidates (TBC1D4, PIK3CD, and VPS37C). Experimental efforts including gene knockdown/knockout (via siRNA or CRISPRi) in PD-relevant models such as iPSC-derived dopaminergic neurons can be applied to assess critical PD phenotypes including α-Syn aggregation, autophagic flux, and phosphorylated α-Syn. Overall, this data-driven approach demonstrates how our findings can directly guide and accelerate future translational efforts and is offered as a reference for further research.

## Methods

### PN Data and Their Functional Interactions.

A comprehensive list of PN was obtained from the Proteostasis Consortium (59, 60) (https://www.proteostasisconsortium.com/). Functional pairwise interactions (version 2021) were downloaded from the Reactome database (https://reactome.org/). All predicted interactions were filtered out from this set of pairwise interactions obtained from Reactome.

### Identification of the First-Degree PN Regulators of α-Syn.

To identify the PN proteins that directly exert a functional interaction on α-Syn, we filtered the pairwise interactions for any inward functional interactions from any protein within the Proteostasis Consortium’s list of PN proteins with α-Syn. The unique list of proteins obtained from the one-directional edgelist resulted in a set of first-degree α-Syn interactors (also referred to as primary α-Syn PN) visualized in Fig. 1. To ensure relevance of the first-degree PN proteins found, we carried out a literature search for evidence supporting their regulatory roles in α-Syn aggregation (Dataset S1). Based on the reports found in literature, we further classified them into promoters and attenuators of α-Syn aggregation.

### PD Microarray Datasets and Consistently Perturbed Genes Relative to *SNCA*.

To identify a consensus set of genes whose expression in relation to *SNCA* is altered in PD compared to controls, 6 PD microarray datasets from the NCBI Gene Expression Omnibus (www.ncbi.nlm.nih.gov/geo/): GSE8397 (61), GSE7621 (62), GSE20163 (63), GSE20292 (63), GSE20333, and GSE43490 (64) were downloaded and analyzed. These datasets were selected as they had minimally five control and five disease samples from the *substantia nigra*. The differential analysis between disease and control samples was performed separately for each dataset before the results were pulled together for further comparison. All expression values were log2-transformed. To calculate the relative expression of each gene to *SNCA* (for evaluating balance of proteins with respect to α-Syn), we normalized all genes to *SNCA* expression by taking log2(gene)-log2(*SNCA*). Statistical differences between the relative expressions (gene:*SNCA*) between groups were evaluated using the two-sided Wilcoxon test with Bonferroni correction for false-discovery detection. A cutoff of absolute fold change > 0.3 and FDR < 0.05 was used. Based on these thresholds, 108 genes were found to be consistently altered (directionality) in at least half of the datasets analysed (Dataset S2).

### Incorporating Consensus Genes into the α-Syn PN.

Seeking to incorporate the relevant subset of the 108 consensus genes into the α-Syn PN, we looked for pairwise functional interactions between each of the 108 genes with the existing primary first-degree PN interactors of α-Syn. From here, 21 genes were found to have functional interactions with the first-degree primary α-Syn PN established earlier and were included in the final α-Syn PN (Dataset S3). These 21 genes were determined to be upstream genes potentially capable of regulating the α-Syn PN and were hence added into the α-Syn PN. Inward functional interactions between each of the 108 genes and α-Syn itself were also searched for. However, none of the 108 genes were found to exert inward functional activities on α-Syn within this dataset.

### Validating the 21 Upstream Genes within the α-Syn PN.

To validate our consensus gene set, we downloaded an additional microarray dataset GSE49036 (65) which had information on Braak staging for comparison. Similar to the procedure described above, all expression values were log2-transformed, normalized to *SNCA*, and visualised in a boxplot by Braak staging.

### PAS.

We define the PAS in a similar way to the subnetwork expression metric used in the context of protein-interaction networks (66). The metric over the network of size K is defined as,

$$\mathrm{PAS}=\frac{1}{\sqrt{K}}\sum_{i\in N}a_{i}z_{i},$$

where $z_{i}$ denotes the z-score normalized expression profile of gene *i* across all genes and $a_{i}$ is the pathway activation sign with $a_{i}$ = 1 if activating and $a_{i}$ = −1 if inactivating/inhibitory.

### Influence Scores of Primary α-Syn PN Components.

To quantify the relative importance of each protein within the α-Syn PN, the influence score of each protein was used as a proxy for its importance within the network. The heat diffusion (39) and PPR (40) algorithms were applied to all proteins within the α-Syn PN network to estimate their influence. The influence scores are available in Dataset S4.

### Nondisease Multiple Brain Region Datasets.

Two human datasets including nondisease samples from various brain regions were used to study the association of our genes with regional vulnerability to PD: UKBEC (38) and GSE7307. Six Braak-stage related regions were delineated R1–R6 (of increasing resilience/decreasing vulnerability) as previously reported (25): R1—myelencephalon, R2—pons, R3—*substantia nigra*, R4—amygdala, R5—temporal cortex/ACC, and R6—frontal cortex. Samples from brain regions R1, R3, R5, and R6 were available within UKBEC and downloaded for analysis. The biomaRt package was used to map the Affymetrix probe IDs from the UKBEC dataset to gene symbols in R. Where multiple probes mapped to the same gene, the probe with the highest variance across samples was chosen. All expression values were expressed on the log2-scale and normalized to *SNCA* expression sample-wise as described earlier. Relative expression values were compared and visualized by brain regions.

### A Digital Twin of the α-Syn PN in Human Cells in the *Substantia Nigra*.

Aiming to accelerate the discovery of PD-related mechanisms and drug testing, we created a virtual replicate of the α-Syn PN. For this purpose, we built a digital twin of the α-Syn PN in the context of the human *substantia nigra*. This was done with the goal of enabling simulated experimentation on complex biological processes to gain insights into disease mechanisms and evaluating potential treatments and their effects on biological networks. By forecasting how individual components of the network would change given perturbations, our digital twin would be useful in assisting in translational applications such as target identification and drug discovery.

To build the digital twin of the α-Syn PN in the context of the human *substantia nigra*, single-cell transcriptomic data from *substantia nigra* brain samples were obtained from the human single-cell atlas of the *substantia nigra* (67) (GSE140231). Single-cell gene expression data were processed using the Seurat package (68) in R. The z-score normalization was applied to normalize gene expressions across all genes in each cell. Z-scores for the α-Syn PN components were extracted and used to learn the dependencies between components of our α-Syn PN via their partial correlations by fitting graphical Gaussian models using the GeneNet package in R (https://cran.r-project.org/web/packages/GeneNet/index.html). This method was previously reported to perform better than other methods in predicting network structure and dependencies from gene expression data (69, 70). The resultant dependencies were filtered using qval < 0.05 and used to fit a linear model allowing the prediction of expression changes for each gene in the network given perturbations to a query gene. We note that all single-cells within the dataset that passed quality control were used for training and not differentiated for cell type. Accordingly, the current digital twin captures features of the *substantia nigra* at a general cellular level, rather than modeling a specific cell type.

### Gene Expression Perturbation Experiment Simulation.

To simulate knockdown and overexpression experiments, we modulated the expression value of each query gene by the following—knockdown: 0.06, 0.12, 0.25, 0.5, 0.75 times the basal level, and overexpression: 1.25, 1.5, 1.75, 2, 4, 8 times the basal level in each cell. We then predicted new gene expression levels for the other genes within the network. Using the new expression levels, we calculated an updated PAS for each cell. For visualisation in Fig. 3, gene downregulation samples were gene expression levels given perturbation of a query gene at 0.12 times the basal level, while overexpression was gene expression levels given perturbation of a query gene at 8 times the basal level in each cell. Detailed simulated data at varying concentrations of downregulation and overexpression for known benchmark targets and our top 3 potential targets are available in *SI Appendix*, Fig. S3.

### Comparison and Selection of Cell Line Models for Drug Testing.

nTPM gene expression values for 1,206 cell lines were obtained from the HPA (https://www.proteinatlas.org/). Cell lines were filtered for *SNCA* nTPM > 2 to ensure detectable *SNCA* expression. The resulting 631 cell lines with detectable *SNCA* expression were profiled for their PAS as follows. To obtain relative gene expression levels, we calculated the ratio of all genes to *SNCA*. Next, z-score normalization was applied to the relative gene expression ratios. The gene expression levels for genes within the α-Syn PN were extracted for each cell line and a corresponding PAS was calculated. Finally, a z-score normalization was applied to normalise PAS across all cell lines. A p-value was also calculated, with *P*-value < 0.05 taken as the cutoff for significance (30 cell lines). The cell lines were ranked in descending order from highest to lowest PAS, prioritizing cell lines with relatively more α-Syn promoting PNs. Thus, selection implies measurable *SNCA* and broad α-Syn PN activity representation in selected lines.

### LINCS Dataset.

CMAP LINCS 2020 level 5 data (level5_beta_trt_cp_n720216x12328.gctx) was downloaded from clueio (https://clue.io/data/CMap2020#LINCS2020). Only data from HEK293 and Jurkat cells treated with small molecules were extracted for use in this study. For each condition (small molecule treatment at a given concentration), the change in PAS (small molecule treatment vs. DMSO/control) was calculated to understand the effects of the small molecule on the α-Syn PN. The change in PAS (ΔPAS) was defined as:

$$\Delta \mathrm{PAS}=\frac{1}{\sqrt{K}}\sum_{i\in N}a_{i}e_{i},$$

where $e_{i}$ denotes the differential relative expression (differential expression of gene_i_:*SNCA*) of a gene *i* while $a_{i}$ is the pathway activation sign with $a_{i}$ = 1 if activating and $a_{i}$ = −1 if inactivating/inhibitory for the α-Syn PN sized K.

For each of HEK293 and Jurkat cell models, the correlation between the increasing concentration of each drug (log scale) and ΔPAS was calculated. A *P*-value < 0.05 and absolute correlation score of 0.5 was used as cutoff. Drugs with negative correlation scores were classified as inhibitors, shifting the activity of the α-Syn PN toward preventing α-Syn aggregation. In contrast, drugs with positive correlation scores were calculated as promoters, shifting the activity of the α-Syn PN toward promoting α-Syn aggregation.

### Drug Prioritization.

For repurposing drugs for PD, we prioritized drugs with inhibitory effects on α-Syn aggregation via the α-Syn PN. Of the 813 drugs tested in both cell lines, 28 drugs were found to consistently exhibit inhibitory effects on α-Syn aggregation via the α-Syn PN. These drugs were ranked based on their average absolute correlation scores in each cell line.

## Supplementary Material

Appendix 01 (PDF)

Dataset S01 (XLSX)

Dataset S02 (XLSX)

Dataset S03 (XLSX)

Dataset S04 (XLSX)

## Data Availability

All data are included in the article and/or supporting information. The code used for the calculation of pathway activity scores is available at https://github.com/christinemarialim/aSyn_PN.git (71).
