# Supplementary material for: The α-synuclein proteostasis network and its translational applications in Parkinson’s disease
Source: Proc Natl Acad Sci U S A. 2026 Mar 16;123(12):e2513317123. doi: 10.1073/pnas.2513317123 (PMC13012101; doi:10.1073/pnas.2513317123)
Supplement: Supplementary file 1 — Appendix 01 (PDF) [file pnas.2513317123.sapp.pdf]

SUPPLEMENTARY INFORMATION

The α-Synuclein Proteostasis Network and its Translational Applications in Parkinson's disease

Christine M Lim* and Michele Vendruscolo*

Centre for Misfolding Diseases, Yusuf Hamied Department of Chemistry,
University of Cambridge, Cambridge CB2 1EW, UK

*Corresponding authors: mv245@cam.ac.uk, cml84@cam.ac.uk

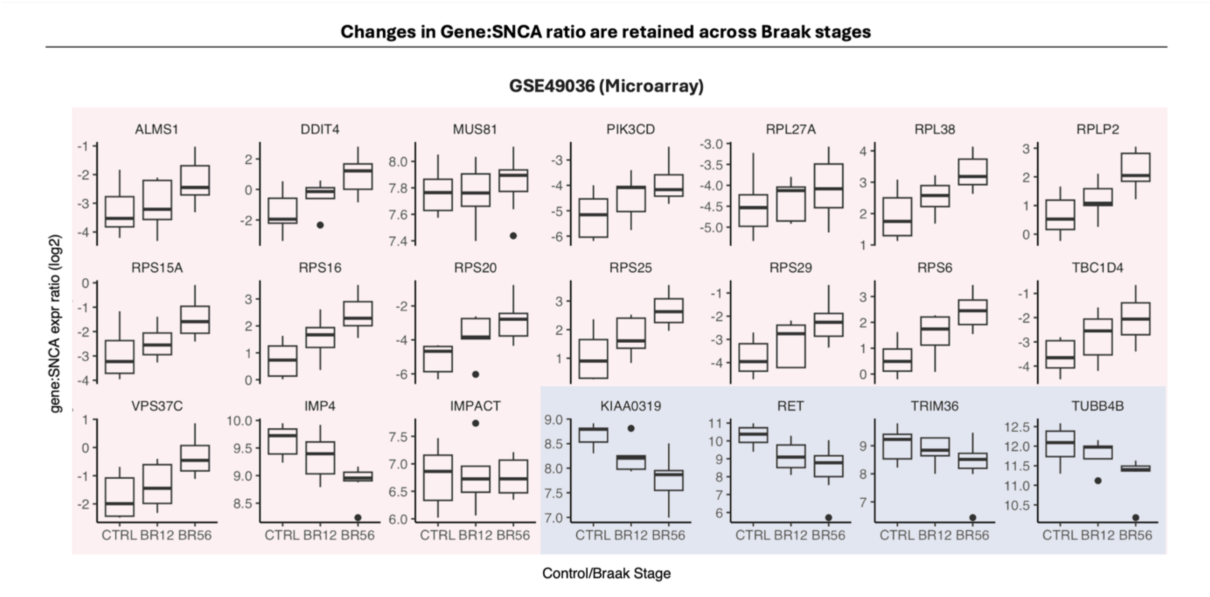

Figure S1. Validating the perturbation trends of network proteins across Braak stages in PD patients. We found 108 genes to be consistently perturbed relative to *SNCA* in PD brains in majority of the 6 PD patient datasets analysed (**Methods**). Of these 108 genes, 21 were found to encode proteins that have functional interactions with the first-degree primary α-Syn PN **(Dataset S1)**. To validate their perturbation patterns in PD, we analysed GSE49036 which is a microarray dataset consisting of *substantia nigra* samples from PD brains of different Braak stages, finding that the same trends of perturbation are retained **(Dataset S2)**.

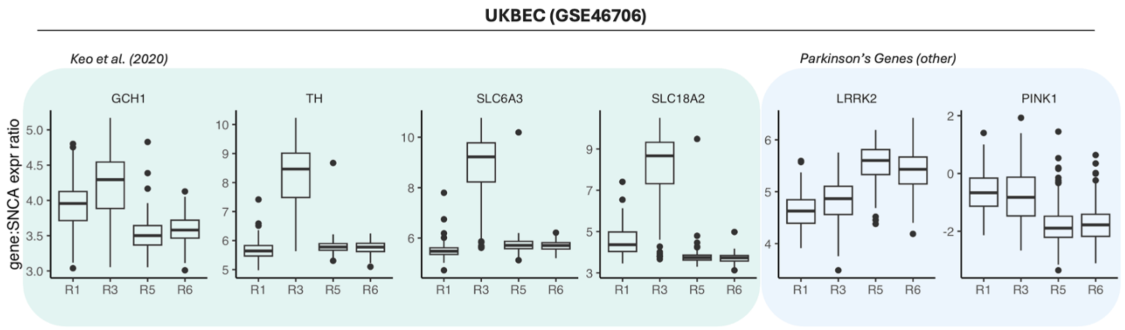

Figure S2: Benchmarking PD-associated genes by relative expression to *SNCA*. Relative expression analysis of known PD genes (e.g., LRRK2 and PINK1) was performed to compare their expression balance against *SNCA* in healthy brain samples across regions of differential vulnerability to PD. The relative expression of these benchmark genes is indicative of regional susceptibility, consistent with previous reports. In contrast, genes identified solely by differential expression without normalization to *SNCA* did not show regionally relevant patterns. These results validate the use of gene:*SNCA* relative expression changes as a strategy to benchmark gene relevance to α-Syn proteostasis and PD vulnerability.

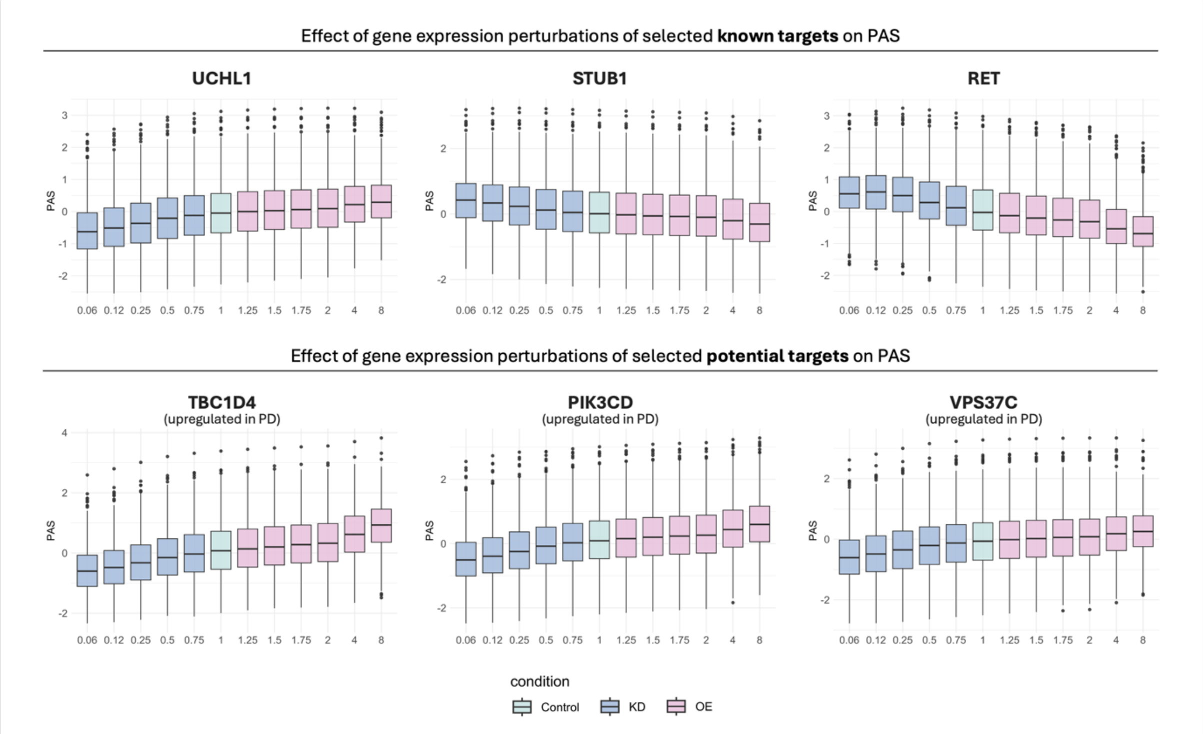

Figure S3. Effect of gene expression perturbations of selected known PD targets and potential PD targets on the activity of the α-Syn PN. To study the modulatory effects potential targets have on the activity of the α-Syn proteostasis network, we simulated the knockdown and overexpression of selected known targets and potential targets to mimic typical knockdown and overexpression experiments done in cells. Details of the simulations are described in **Methods**. By benchmarking our simulations to known targets, we found that overexpression of α-Syn aggregation-promoting UCHL1 increases the aggregation-promoting activity of the α-Syn PN upon upregulation and takes a more inhibitory slant upon downregulation; α-Syn aggregation-inhibiting STUB1 and RET shifts the activity of the α-Syn PN towards inhibiting α-Syn aggregation upon upregulation while causing a slant towards aggregation-promoting activity upon their downregulation. The top 3 potential targets based on their effect size on the α-Syn PN given expression perturbations are TBC1D4, PIK3CD, and VPS37C. All 3 targets are upregulated relative to *SNCA* in PD conditions and are found to pivot the activity of the α-Syn PN towards promoting α-Syn aggregation upon overexpression. In contrast, downregulating these targets shifts the network closer towards α-Syn inhibition. These findings suggests that inhibition of TBC1D4, PIK3CD, and VPS37C may potentially be exploited for managing the shift of the α-Syn PN towards promoting aggregation.
